# Influence of Voice Interactive Educational Robot Combined with Artificial Intelligence for the Development of Adolescents

**DOI:** 10.1155/2022/7655001

**Published:** 2022-10-06

**Authors:** Yadong Zhang, Hongkai Wang

**Affiliations:** ^1^School of Art and Design, Shandong Women's University, Jinan 250000, China; ^2^Academy of Arts and Design, Tsinghua University, Beijing, China; ^3^College of Journalism and Communications, Shih Hsin University, Taipei, China

## Abstract

In the context of multicultural information, to explore and analyze the use effect of voice interactive educational robot in the classroom of adolescent students, and the physical and mental impact of movie characters on adolescent students, and to lay the foundation for studying the positive development of adolescents, under the guidance of positive psychology theory, the relationship between positive psychology and adolescent mental health is analyzed, the application of adolescent educational robot is discussed, and the relationship between adolescent educational robot combined with movies characters and positive development of adolescent is analyzed. The questionnaire is used to collect data, including the questionnaire on the influence of movie characters on the positive development of adolescents, the questionnaire of pre- and postpopularization of artificial intelligence in primary and secondary schools, and the satisfaction questionnaire of voice interactive educational robot. The reliability and validity of the questionnaire are analyzed. The results show that students who watch more than 20 movies are 20% in grade one and that is only 10% in grade three. 79% of the students think that the movie characters have an impact on themselves. The distribution of the number of the grade two students exposed to the movie is relatively uniform, 40% of them watch 10-20 movies, and 82% of them think that the movie characters have an impact on themselves; the Cronbach coefficient of classroom satisfaction questionnaire is 0.929 > 0.9, the average value of the correlation of total correction items of the corresponding item is 0.612 > 0.5, the Kaiser–Meyer–Olkin measurement value is 0.812 > 0.6, and the Sig value is 0.000 < 0.05, indicating that the reliability and validity of the questionnaire are very high and 92.3% of the students are very satisfied with the classroom. This shows that voice interactive educational robot combined with movie characters can promote the positive development of adolescents.

## 1. Introduction

The development of human society is becoming more and more complicated. In the case of rapid multiparty flow of information, the speed of cultural renewal is also greatly improved. Meanwhile, different cultures need to serve the development of the society, which gradually forms a multiculture [1, 2]. Adolescents are the hope of national development, and they should grow and develop in a multicultural background. The common psychological problem of teenagers is the inferiority complex [3]. Most people have had inferiority complex in a certain period of growth or under certain circumstances. A slight sense of inferiority complex can promote people's discovery and improvement of their shortcomings, but excessive inferiority complex will make people lose hope for life, produce negative self-definition, and ultimately have adverse consequences [4]. Effective psychological intervention can help schools and families to pay attention to the harm of adolescents' inferiority complex, which can help adolescents establish self-confidence and make them in a healthy state of development. At present, foreign countries have attached great importance to the psychological problems caused by the sense of inferiority of adolescents. Most of the domestic studies in related fields focus on the theoretical aspect. In fact, only a few of them have taken intervention measures against the sense of inferiority of adolescents, and they have not intervened according to the theory of positive psychology [5].

Information diversity makes society in the information explosion period, and people are surrounded by information all the time. Because of the particularity of the physical and mental environment, adolescents belong to the special group in the media information environment and are vulnerable to the impact of media information, which has an impact on the positive personality development of adolescents [6]. The movies are the core product of the media environment, which has a great impact on the thinking and behavior of people and adolescents. Adolescents are in a psychological state of trust for movie information, but they are also in a passive state. In the face of complex movie types, how should adolescents choose movies that are beneficial to their development, and under what conditions do they internalize the information conveyed by the movie characters? These are the problems that need to be solved when movies are selected for adolescents [7]. With the progress of science and technology, while media information is used to develop education on adolescents, the cultivation of artificial intelligence talents has occupied the core part of China's education. Adolescents are the reserve force of national science and technology progress. The artificial intelligence enlightenment education of adolescents is highly valued by the education department. The incorporation of artificial intelligence education into the classroom of adolescents will provide hope for the improvement of the national education level [8]. Artificial intelligence education in foreign countries starts earlier, and the artificial intelligence education in the United States has entered the national strategic stage, which strongly supports the relevant funds, policies, and talent introduction. Adolescent artificial intelligence education in Australia is a compulsory course. Adolescents will study the theoretical knowledge of artificial intelligence in the way of discussion and experimental operation [9, 10]. In the current adolescent education in China, the use of multimedia classrooms and the information-based training of educators show that artificial intelligence education has gradually replaced the traditional teaching mode and promoted the leap from theory to practice stage of adolescent education [11].

Based on this, under the guidance of positive psychology theory, questionnaire survey and empirical analysis are used to study the use of voice interactive robot in the classroom of adolescents and the influence of movie characters on the physical and mental development of adolescents, which can provide a practical basis for promoting the positive development of adolescents.

## 2. Method

### 2.1. Positive Psychology and the Development of Adolescents

Adolescents are in a critical period of physical and mental development. Positive psychology is used to help adolescents overcome inferiority complex and form a positive self-awareness concept, which can greatly promote the mental health level and positive development of adolescents [12].

#### 2.1.1. Theoretical Basis of Positive Psychology

The research content of positive psychology includes positive emotional feelings such as full of love and subjective well-being, and how to obtain positive feelings and healthy mental state [13]. In positive psychology, it is proposed that positive emotional feeling can make an individual's work and life full of positive energy. According to the theory of “Broaden-and-Build” [14], even the unsystematic positive emotion can change the individual's instantaneous behavior ability and also improve the individual's intelligence quotient, social adaptability, and physical strength. Positive emotional experience can also actively prevent diseases and can promote the body's immunity to diseases. In positive psychology, it is also pointed out that the external and cultural environment will change the mental health level of individuals in varying degrees. When the positive personality characteristics are established, the individual also establishes a positive environment system. It means that positive psychology starts from the factors of individual growth environment and promotes individual to form positive personality by establishing positive external environment, family environment, and campus environment.

#### 2.1.2. The Relationship between Positive Psychology and Mental Health of Adolescents

In mental health education, by observing the age and physical and mental development characteristics of adolescents and using various methods and measures, the mental potential of adolescents has been continuously explored, and their ability to resist pressure has been improved, which makes the physical and mental health of adolescents develop harmoniously [15]. As the main field of learning and life of adolescents, school mental health education belongs to the practical influence range of psychology. Under the influence of positive psychology, the teaching methods of school mental health education are constantly changing. In the past, school mental health education focused on the prevention and treatment of students' mental problems, and the measures taken were mainly mental education courses and psychological counseling, which can relieve the pressure and distress of adolescent students [16]. The positive mental health education starts from the positive elements of the students and guides the students to pay attention to their advantages, to be good at paying attention to the positive energy around them and to cultivate their self-confidence, which can produce a sense of self-efficacy, and make them develop in an all-round way while paying attention to their spiritual needs.

### 2.2. Artificial Intelligence and Active Development of Adolescents

In artificial intelligence, machines are used as the intermediate carrier to complete the simulation of human work and life [17]. Its core part is human intelligence. First, the brain is used as the intermediary to generate the ability of memory, research, and exploration; second, the perception organ carries out the perception of external stimulation; finally, the mouth, arm, and other parts are used for quick information feedback. As a part of computer science, artificial intelligence has a great impact on the application and development of information technology [18].

#### 2.2.1. Theoretical Basis of Educational Robot

The important purpose of the educational robot is to assist classroom education. The aim of artificial intelligence educational robot is people-oriented. Developmental toys can be used as an auxiliary tool for the teaching of basic education courses in schools. It can be used to educate students from various directions and finally realize “education through entertainment.” Educational robots have the immeasurable potential in the process of education, which can make the educational cause have subversive changes. The object of the educational robot is students, and it can program students' behavior to achieve the purpose of assisting teaching. The robot-assisted teaching is the activity of teaching and learning with robot as a teaching aid tool. From the perspective of sociology [19], the educational robot can play the role of teacher and classmate and complete corresponding tasks; from the perspective of communication studies [20], it can expand the dissemination of teaching information, improve educators' knowledge reserve, and reduce students' learning pressure; based on the perspective of educational psychology, the educational robot can promote students' learning behavior and make students' knowledge perception and comprehensive utilization ability be improved. At present, the teaching forms of the educational robot are various, including the auxiliary of giving and listening to lessons, answering questions, teaching, and learning games.  Table 1 shows the classification of educational robots.

#### 2.2.2. The Relationship between Voice Interactive Educational Robot and the Development of Adolescents

**Figure 1:**
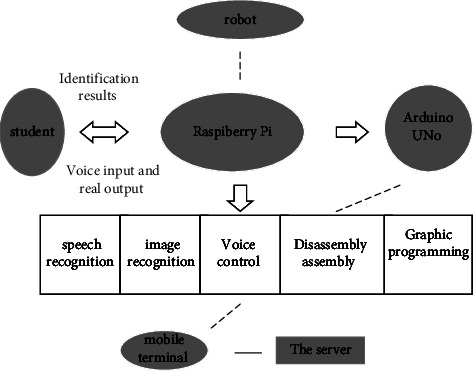
Structural framework of voice interactive educational robot.

The voice interactive robot can use a computer carrier to transform speech content into text. The goal is to realize the language conversation of the machine, so that the machine can understand the meaning of human speech [21]. Hidden Markov model is used; feature extraction, pattern matching, and model training technologies are combined; and the theoretical model of digital signal processing is mixed with many disciplines such as phonetics [22]. For the voice recognition of the current voice interactive robot, a great breakthrough has been made in the defects of accent, speech speed, and noise influence, which greatly promotes the education of adolescent students. The main application objects of the voice interactive educational robots are college students and adolescent primary and secondary school students. Its goal is to improve the scientific literacy of college students and adolescents. For adolescent students, improving their innovative thinking is the key purpose, which can greatly stimulate students' interest in learning. The application principle of voice interactive educational robot is the educational concept of “education through entertainment.” It is used in language and behavior programming to achieve the purpose of educational companion, which is widely favored by adolescent learners. Many domestic digital learning equipment manufacturing enterprises, such as Sang Technology company, are committed to the research and development of voice interactive educational robots and corresponding teaching platforms, instilling the concept of robot education into primary and secondary school curriculum education. Through the synthesis of artificial intelligence thought and artificial intelligence knowledge, the voice interactive educational robot realizes the auxiliary function of artificial intelligence teaching, completes the perfect integration of technology and knowledge, creates a real artificial intelligence environment for the adolescent students, and helps them to explore the artificial intelligence world preliminarily by applying colorful activities to develop the students' intelligence to the greatest extent.  Figure 1 shows the structure of the voice interactive educational robot.

### 2.3. Movie Characters and the Positive Development of Adolescents

#### 2.3.1. Theoretical Basis

The core concern of positive psychology is the research on the individual's development potential and positive personality traits, which is a newly developed discipline [23]. Because of its special psychological characteristics, adolescents are in a special period of mental health education. Therefore, the combination of positive psychology and mental health education of adolescents has become a new direction of the development of education in China. At the same time, the corresponding research on the positive development of adolescents has gradually attracted attention. The positive development of adolescents was first put forward by Little in 1993. After continuous theoretical and practical improvement, it has formed a psychological theory with the development of adolescents based on the positive attitude as the core concern. In American encyclopedia, it is proposed that positive development provides a theoretical basis for schools to improve students' interest in learning and learning ability. Its core concern is to provide a supportive environmental atmosphere for adolescents and to provide practical exercises for adolescents through different channels, which make adolescents have the courage to contribute to the society. In addition, a positive attitude is adopted to pay attention to the development of adolescents to help them find their advantages and disadvantages as well as their learning ability, so that their advantages can be developed to the maximum extent. Effective methods are explored to develop the advantages of adolescents and encourage them to actively participate in meaningful activities. Therefore, the emergence of positive psychology can make adolescents focus on their positive aspects and not pay too much attention to their negative aspects and turn the adolescent education view to the education that promotes the potential of adolescents [24].

Bandura proposed that the ability to learn through observation can harvest complex and unified holistic behaviors, and it is not necessary to get correct conclusions in the process of constantly trying and making mistakes [25]. Individuals can internalize and learn by observing other people's behaviors in solving problems. People learn only by trying and making mistakes, which is very hard and time-consuming. If their behavior and the corresponding results are observed, learners can correct their behavior without their practice. According to the guidance of observational learning theory, the influence of characters in contemporary movies on the positive quality of adolescents is analyzed.

#### 2.3.2. The Influence of Movie Characters on the Positive Development of Adolescents

**Figure 2:**
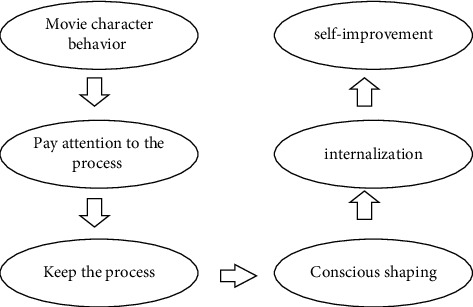
Model of movie's influence on adolescents.

The main function of the movie is to create vivid characters and set their personality charm and show the rich cultural and social characteristics of the modern society in many performance forms to exert its subtle influence [26]. Under the current era background and social trend, the way of expression of the outlook on life, values, and behavior characteristics expressed by movie characters conforms to the characteristics of the times. It can help adolescents to fully understand the beautiful life and society, learn the behavior norms in daily life, and help adolescents release their emotions and learn to release their pressure with entertainment. In the process of watching movies, adolescents do not need to spend too much time adapting to the movie environment and can easily feel the information expressed by the movie characters. At the same time, some information and concepts are transformed into their internal parts, which influence the behavior of adolescents. Therefore, from a certain point of view, movies and movie characters become educational tools outside the campus, which can promote the growth and positive development of adolescents.  Figure 2 shows a model of the impact of movies on adolescents.

At the same time, because of the wide-spread advantages of movies, the positive educational effect of movie characters on adolescents will even exceed the influence of campus education. This can lead to educators' thinking and attention, that is, to introduce movies and movie characters into the campus teaching environment, to stimulate students' learning enthusiasm through the movie's liveliness, and to carry out cultural education under the condition of physical and mental relaxation of adolescents, which plays an important auxiliary role in the positive development of adolescents.

### 2.4. Survey Method

Questionnaires are used to collect data, including the questionnaire on the influence of movie characters on the positive development of adolescents, the questionnaire of pre- and postpopularization of artificial intelligence in primary and secondary schools, and the satisfaction questionnaire of voice interactive educational robot. Then, the information in the questionnaire is statistically analyzed.

1. The questionnaire on the influence of movie characters on the positive development of adolescents consists of two parts. One is the types and attention of adolescents to movie characters; the other is the influence of movie characters on adolescents. XX middle school students are randomly selected as the research object. According to the proportion of boys and girls in the school, a total of 260 questionnaires are distributed. A total of 200 valid questionnaires are selected based on the influence of the movie characters proposed by the students. From the effective questionnaire, 80 students (52 boys and 28 girls) are randomly selected to conduct in-depth interviews on the influence of movie characters. The results of the interview are consistent with the hypothesis of the valid questionnaire.
2. Class 3 students (72 students) in grade two of XX middle school are investigated. According to the Learning and Study Strategies Inventory (LASSI), evaluation of classroom satisfaction of the voice interactive robot (artificial intelligence educational robot car with Raspberry Pi and Arduino as the carrier) is carried out in four directions: teaching method, teaching attention, teaching atmosphere, and teaching effect feedback. For the evaluation of teachers, there are teaching methods and teaching organization. In terms of the students' attention, learning awareness, and behavior, there are 15 questions in total. The questionnaire imitates Likert five-point scale method [27], including five parts: very satisfied, satisfied, general, dissatisfied, and very dissatisfied. The scores can be divided into 5, 4, 3, 2, and 1 in turn, as shown in Tables 2 and 3.

## 3. Results

### 3.1. The Influence of Movie Characters on the Positive Development of Adolescents

**Figure 3:**
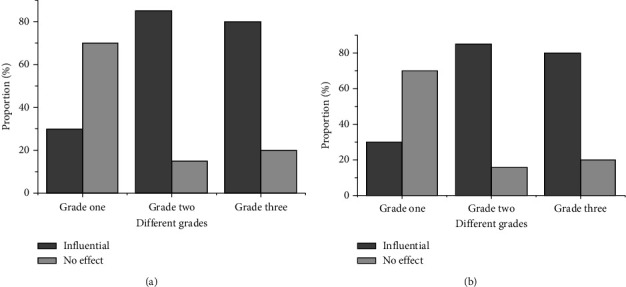
Influence of movie characters on middle school students ((a) the proportion of students in three grades of middle school watching movies; (b) the proportion of students affected by movie characters).

According to the effective questionnaire, in-depth communication on whether the movie characters have an impact on them is conducted. Figures 3(a) and 3(b) show the results.

Figure 3 shows that the grade one students of middle school can contact with movies, but the distribution of contact quantity is uneven. In grade one, the number of students who watch less than 10 movies accounts for 46%, only 20% of them watch more than 20 movies, and only 30% of them think that the movie characters have an impact on themselves; the distribution of the number of students in grade two exposed to the movie is relatively uniform, 40% of them watch 10-20 movies, and 82% of them think that the movie characters have an impact on themselves; the distribution of the number of students exposed to movies in grade three is also uneven, 55% of them watch less than 10 movies, only 10% of them watch more than 20 movies, and 79% of them think that the movie characters have an impact on themselves.

### 3.2. The Influence of Voice Interactive Robot on the Positive Development of Adolescents

#### 3.2.1. Reliability of the Questionnaire

**Figure 4:**
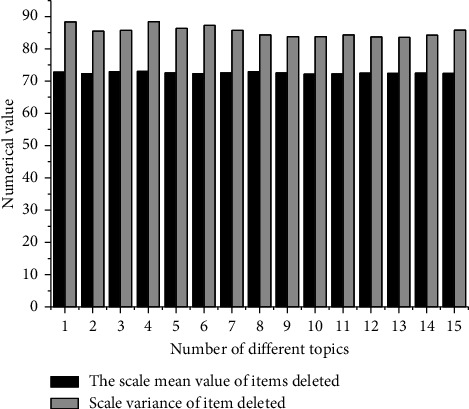
Analysis results of classroom satisfaction questionnaire (the Cronbach coefficient of each item is 0.929, the average value of the correlation of total correction items is 0.612, and the average value of *α* coefficient deleted items is 0.927).

First, the reliability of the questionnaire is analyzed for 72 students who participate in the class, as shown in Figure 4.

Figure 4 shows that the Cronbach coefficient of the classroom satisfaction questionnaire is 0.929 > 0.9, which indicates that the quality of the collected information data is high. Compared with the Cronbach coefficient, there is no obvious change in the *α* coefficient value of the deleted item, so all 15 items in the questionnaire can be used. The average value of the total correlation of the corresponding correction items is 0.612 > 0.5, indicating that the correlation between the analysis items is very good. Therefore, the reliability level of information data is high, and further study can be carried out.

#### 3.2.2. Validity of the Questionnaire

According to the above results, the validity of the questionnaire is analyzed by factor analysis.  Table 4 shows the analysis results.

Figure 4 shows that the Kaiser–Meyer–Olkin measurement value is 0.812 > 0.6, and the Sig value is 0.000 < 0.05, indicating that the classroom satisfaction questionnaire can be used to carry out factor analysis.

**Figure 5:**
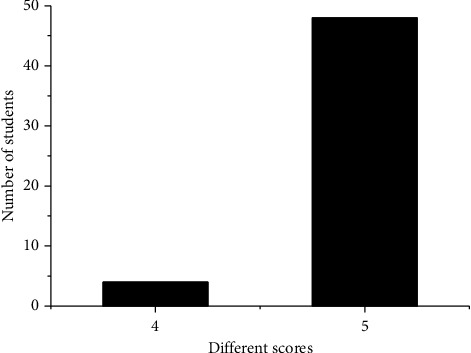
Score of classroom satisfaction questionnaire.

Finally, according to the classroom satisfaction questionnaire, score results are counted, as shown in Figure 5.

Figure 5 shows that there are 48 students with 5 points and 4 students with 4 points. The number of students who get 1, 2, and 3 points is 0, which means that 92.3% of students are very satisfied with the classroom, that is to say, voice interactive robots are welcomed by most students.

## 4. Discussion

Grade one students have just entered middle school, and they are in a strange and curious stage about the school environment. Therefore, they will not put too much energy into the movie. Therefore, the proportion of students who watch more movies is smaller, and the influence of movie characters on them is not strong. Grade two students are in the critical period of adolescents' ideological and moral transformation [28]. Being familiar with the campus environment and learning, they will have a strong curiosity about other things and have time and interest in watching movies. Therefore, the proportion of grade two students who watch more movies is larger because of the particularity of students' physical and mental characteristics [29, 30], and the influence of movie characters on them is also very strong. The grade three students face the pressure of entering school and have no time and energy to watch movies, so the proportion of students who watch more movies is smaller. However, grade three students tend to be mature physically and mentally, so they can distinguish the behavioral characteristics in the movie characters and are easily influenced by the characters, which is consistent with the research results of Hsia et al. [31].

Students are very interested in the human-computer interaction classroom learning mode, and most students show interest in the teacher's teaching method, which is consistent with the research results of Li et al. [32]. Students hope to learn more extensive artificial intelligence knowledge with their classmates on the school, and then use their spare time to understand other knowledge in the field of artificial intelligence [33–38]. Some students hope to carry out the work in the field of artificial intelligence in the future [39–46]. It shows that the voice interactive educational robot under artificial intelligence can promote the positive development of adolescents.

## 5. Conclusion

Under the background of multicultural information, based on the theory of positive psychology, the voice interactive educational robot using artificial intelligence works as the assistant education tool of adolescents. More than 95% of the students are very satisfied with the classroom, and they have a great interest in learning in the classroom. For middle school students, the number of students watching movies in grade two is higher. Even though the proportion of students watching more movies in grade one and grade three is relatively small, the influence of movie characters on students of three grades is relatively great. Therefore, voice interactive educational robot combined with movie characters will promote the positive development of adolescents. However, there are also some deficiencies. The area and number of participants in the questionnaire survey are limited, and the data is lack of certain universality, which needs to be improved in the follow-up study.

## Figures and Tables

**Table 1:** Classification of adolescent educational robots.

Classification	Main function
STEAM teaching aids (Kaizhi KP3, Fischertechnik, and mBot)	Scientific, free to build, physics science popularization, visualization
Classroom-assisted teaching (Keeko intelligent robot)	Language teaching, teaching according to age characteristics, strong sports ability

**Table 2:** Classroom evaluation methods.

Object	Evaluation direction
Teachers	Teaching organization; teaching method
Students	Attention level; learning awareness and behavior

**Table 3:** Setting of classroom satisfaction questionnaire.

Degree	Score
Very satisfied	5 points
Satisfied	4 points
General	3 points
Dissatisfied	2 points
Very dissatisfied	1 points

**Table 4:** Results of validity analysis of classroom satisfaction questionnaire.

Different parameters	Numerical value
Kaiser–Meyer–Olkin measurement	0.812
Approximate chi square	1050.135
D*f*	14
Sig	0.000

## Data Availability

The raw data supporting the conclusions of this article will be made available by the authors, without undue reservation.
